# Paediatric leukaemia DNA methylation profiling using MBD enrichment and SOLiD sequencing on archival bone marrow smears

**DOI:** 10.1186/s13742-015-0050-0

**Published:** 2015-03-18

**Authors:** Nicholas CL Wong, Gavin D Meredith, George Marnellos, Miroslav Dudas, Mandy Parkinson-Bates, Minhee Suh Halemba, Zac Chatterton, Jovana Maksimovic, David M Ashley, Francoise Mechinaud, Jeffrey M Craig, Richard Saffery

**Affiliations:** 1 Cancer and Disease Epigenetics Research Group, Murdoch Childrens Research Institute, Royal Children’s Hospital, Flemington Road, Parkville, 3052 Victoria Australia; 2 Department of Paediatrics, The University of Melbourne, Children’s Hospital, Flemington Road, Parkville, 3052 Victoria Australia; 3 Translational Genomics and Epigenomics Research Group, Ludwig Cancer Research, Olivia Newton-John Cancer and Wellness Centre, Austin Hospital, Burgundy Street, Heidelberg, 3084 Victoria Australia; 4 Andrew Love Cancer Centre, Deakin Universit, Swanston Street, Geelong, 3220 Victoria Australia; 5 Children’s Cancer Centre, Royal Children’s Hospital, Flemington Road, Parkville, 3052 Victoria Australia; 6 Early Life Epigenetics Research Group, Murdoch Childrens Research Institute, Royal Children’s Hospital, Flemington Road, Parkville, 3052 Victoria Australia; 7 Life Technologies, Carlsbad, 92008 CA USA; 8 Bioinformatics Group, Quantitative Sciences Core, Murdoch Childrens Research Institute, Royal Children’s Hospital, Flemington Road, 92008, Parkville, 3052 Victoria Australia; 9 Pacific Edge Limted, 84 St David Street, Dunedin 9016, New Zealand; 10 Current Address: Science Division Informatics Group, Faculty of Arts and Sciences, Harvard University, MA 02138, Cambridge USA

**Keywords:** Childhood leukaemia, DNA methylation, SOLiD MBD-Seq, NGS, Epigenetics

## Abstract

**Background:**

Acute Lymphoblastic Leukaemia (ALL) is the most common cancer in children. Over the past four decades, research has advanced the treatment of this cancer from a less than 60% chance of survival to over 85% today. The causal molecular mechanisms remain unclear. Here, we performed sequencing-based genomic DNA methylation profiling of eight paediatric ALL patients using archived bone marrow smear microscope slides.

**Findings:**

SOLiD™ sequencing data was collected from Methyl-Binding Domain (MBD) enriched fractions of genomic DNA. The primary tumour and remission bone marrow sample was analysed from eight patients. Four patients relapsed and the relapsed tumour was analysed. Input and MBD-enriched DNA from each sample was sequenced, aligned to the hg19 reference genome and analysed for enrichment peaks using MACS (Model-based Analysis for ChIP-Seq) and HOMER (Hypergeometric Optimization of Motif EnRichment). In total, 3.67 gigabases (Gb) were sequenced, 2.74 Gb were aligned to the reference genome (average 74.66% alignment efficiency). This dataset enables the interrogation of differential DNA methylation associated with paediatric ALL. Preliminary results reveal concordant regions of enrichment indicative of a DNA methylation signature.

**Conclusion:**

Our dataset represents one of the first SOLiD™MBD-Seq studies performed on paediatric ALL and is the first to utilise archival bone marrow smears. Differential DNA methylation between cancer and equivalent disease-free tissue can be identified and correlated with existing and published genomic studies. Given the rarity of paediatric haematopoietic malignancies, relative to adult counterparts, our demonstration of the utility of archived bone marrow smear samples to high-throughput methylation sequencing approaches offers tremendous potential to explore the role of DNA methylation in the aetiology of cancer.

## Data description

This project was approved by the Royal Children’s Hospital Human Research Ethics Committee (RCH HREC# 29140C). We have performed Methyl-Binding Domain protein 2 (MBD2) enrichment and isolated fractions of DNA from 40 individuals for sequencing on the Sequencing by Oligonucleotide Ligation and Detection (SOLiD™) sequencing platform (SOLiD™MBD-Seq, Life Technologies, Carlsbad, USA). MBD2 has been shown to bind to double-stranded methylated DNA molecules and used to interrogate the human methylome [1]. By comparing the enriched fraction to the "input" total genomic DNA fraction, genomic regions of DNA methylation can be inferred after sequencing both fractions. The samples analysed are comprised of the following: three model cell lines, JWL (an in-house non-leukaemic cell line [2]), CEM-CCRF (childhood T-cell acute lymphoblastic leukaemia [ALL] cell line) and K562 (adult chronic myelogenous leukaemia cell line). From two non-leukaemic individuals (pbsc1 and pbsc2), peripheral blood mononuclear cells were sampled and four haematopoietic cell populations (CD34-positive, CD19-positive, CD33-positive and CD45-positive) were isolated for SOLiD™MBD-Seq analysis. From another two non-leukaemic individuals (bm9 and bm10), the same haematopoietic cell populations were isolated from bone marrow. Eight cases of childhood ALL were analysed with the identifiers 135, 197, 292, 316, 362, 367, 378 and 386 at diagnosis (leuk) and 28 days post induction chemotherapy (rem). A third set of samples was taken at relapse (lap) for cases 197, 316, 362 and 367 (Table 1).

**Table 1:** **Samples analysed in this study and sequencing metrics**

Sequencing chemistry	Sample	TotalTags	UniqueTags	Alignment efficiency
SOLiD v3	JWL(1ug)-E	82,825,332	25,878,330	31.24%
	JWL(5ug)-E	41,496,636	16,004,583	38.57%
	CEM-CCRF-E	70,843,054	23,576,689	33.28%
	K562-E	67,818,656	19,407,273	28.62%
	Leuk316-E	86,478,570	29,043,378	33.58%
	Lap316-E	74,223,311	19,719,258	26.57%
	Rem316-E	77,702,366	23,783,043	30.61%
	NB-Leuk	84,461,471	44,789,628	53.03%
SOLiD v4	bm9_cd19-E	38,269,892	22,810,020	59.60%
	bm9_cd19-I	37,784,067	34,877,526	92.31%
	bm9_cd33-E	10,416,743	6,266,457	60.16%
	bm9_cd33-I	42,759,158	39,428,521	92.21%
	bm9_cd34-E	51,318,758	29,465,956	57.42%
	bm9_cd34-I	46,036,938	42,428,715	92.16%
	bm9_cd45-E	12,914,609	9,361,359	72.49%
	bm9_cd45-I	33,483,409	31,189,190	93.15%
	bm10_cd19-E	45,846,788	23,820,768	51.96%
	bm10_cd19-I	42,916,292	39,433,442	91.88%
	bm10_cd33-E	18,881,635	12,678,559	67.15%
	bm10_cd33-I	36,392,688	33,622,173	92.39%
	bm10_cd34-E	1,455,904	976,835	67.09%
	bm10_cd34-I	43,740,869	38,954,344	89.06%
	bm10_cd45-E	47,832,605	31,267,133	65.37%
	bm10_cd45-I	56,272,359	51,839,725	92.12%
	Lap197-E	23,613,069	17,771,922	75.26%
	Lap197-I	40,839,842	37,160,823	90.99%
	Lap316-E	22,312,029	18,406,671	82.50%
	Lap316-I	65,985,869	59,587,559	90.30%
	Lap362-E	26,103,269	18,291,098	70.07%
	Lap362-I	43,663,529	39,464,174	90.38%
	Lap367-E	30,436,848	22,390,188	73.56%
	Lap367-I	61,426,571	55,393,215	90.18%
	Leu135-E	28,518,319	22,184,998	77.79%
	Leu135-I	66,384,953	59,496,167	89.62%
	Leu197-E	40,781,905	21,042,700	51.60%
	Leu197-I	100,952,576	88,986,493	88.15%
	Leu292-E	37,383,290	27,245,488	72.88%
	Leu292-I	81,946,813	73,469,308	89.65%
	Leu316-E	19,691,035	15,476,011	78.59%
	Leu316-I	49,443,957	44,399,793	89.80%
	Leu362-E	26,155,137	19,508,962	74.59%
	Leu362-I	52,718,699	47,588,023	90.27%
	Leu367-E	30,436,848	22,390,188	73.56%
	Leu367-I	61,426,571	55,393,215	90.18%
	Leu378-E	37,963,480	25,159,304	66.27%
	Leu378-I	63,946,621	57,452,718	89.84%
	Leu386-E	34,541,207	26,730,838	77.39%
	Leu386-I	85,783,795	76,829,088	89.56%
	pbsc1_cd19-E	28,300,798	17,236,825	60.91%
	pbsc1_cd19-I	42,994,203	39,873,845	92.74%
	pbsc1_cd33-E	28,441,237	17,222,149	60.55%
	pbsc1_cd33-I	41,190,719	38,084,036	92.46%
	pbsc1_cd34-E	30,595,326	18,228,854	59.58%
	pbsc1_cd34-I	40,582,296	37,607,618	92.67%
	pbsc1_cd45-E	21,807,673	11,901,508	54.57%
	pbsc1_cd45-I	44,739,461	41,071,013	91.80%
	pbsc2_cd19-E	35,937,656	20,893,976	58.14%
	pbsc2_cd19-I	39,678,926	36,769,939	92.67%
	pbsc2_cd33-E	35,344,009	22,387,891	63.34%
	pbsc2_cd33-I	32,507,100	30,204,900	92.92%
	pbsc2_cd34-E	25,845,401	13,736,296	53.15%
	pbsc2_cd34-I	48,706,315	44,827,413	92.04%
	pbsc2_cd45-E	32,212,432	21,627,452	67.14%
	pbsc2_cd45-I	47,235,290	43,581,366	92.26%
	Rem135-E	36,998,278	24,794,225	67.01%
	Rem135-I	123,775,359	108,611,090	87.75%
	Rem197-E	32,669,979	20,692,190	63.34%
	Rem197-I	72,248,569	64,234,959	88.91%
	Rem292-E	40,308,561	29,524,878	73.25%
	Rem292-I	56,187,553	50,646,668	90.14%
	Rem316-E	29,052,098	23,634,221	81.35%
	Rem316-I	60,566,396	54,693,711	90.30%
	Rem362-E	30,583,568	24,759,911	80.96%
	Rem362-I	59,138,768	53,532,111	90.52%
	Rem367-E	27,854,950	20,285,938	72.83%
	Rem367-I	53,616,042	48,017,388	89.56%
	Rem378-E	19,428,372	14,820,513	76.28%
	Rem378-I	58,333,370	51,624,942	88.50%
	Rem386-E	26,909,681	19,355,786	71.93%
	Rem386-I	83,504,227	74,547,836	89.27%
	Total	3,671,922,955	2,741,473,297	74.66%

Genomic DNA from archived bone marrow smear microscope slides from ALL patients, cells and cell lines were extracted as previously described [3] and used for the enrichment of CpG methylation with the MethylMiner™ Methylated DNA enrichment kit (Life Technologies) according to the manufacturer’s protocols. The fragmented input genomic DNA (I) and enriched E5 fraction (E) were isolated from each sample for library preparation and sequencing using SOLiD™ v3 and v4 chemistry according to the manufacturer’s protocols (Life Technologies).

Single and paired-end SOLiD™ sequencing reads were aligned using LifeScope™ Genomic Analysis Suite (Life Technologies) with default parameters against the hg19 reference genome. Alignment efficiency (the ratio of uniquely aligned reads to total sequenced reads for each sample) ranged from 26.57% to 93.15% across all samples in this study (Table 1).

Alignments were then processed using MACS (Model-based Analysis for ChIP-Seq) [4] and HOMER (Hypergeometric Optimization of Motif EnRichment) [5,6] to identify enrichment peaks.

This study is unique in a number of ways. This is the first sequencing-based DNA methylation profiling study in childhood ALL using archived bone marrow samples of similar quality to formalin-fixed paraffin-embedded (FFPE) tissue samples [7]. We have selected samples that have been interrogated using an orthogonal platform, the Illumina Infinium Human Methylation 450K BeadArray [3,8], and included replicate samples to assess the reproducibility of SOLiD™MBD-Seq and to identify regions of differential DNA methylation of interest to childhood ALL.

We performed replicate DNA methylation enrichment analysis using the JWL cell line with 1 *μ*g and 5 *μ*g of starting genomic DNA to determine if 1 *μ*g of starting material was sufficient for DNA methylation enrichment. This was less than the recommended quantity but a typical amount obtainable from our primary patient samples.

**Figure 1:**
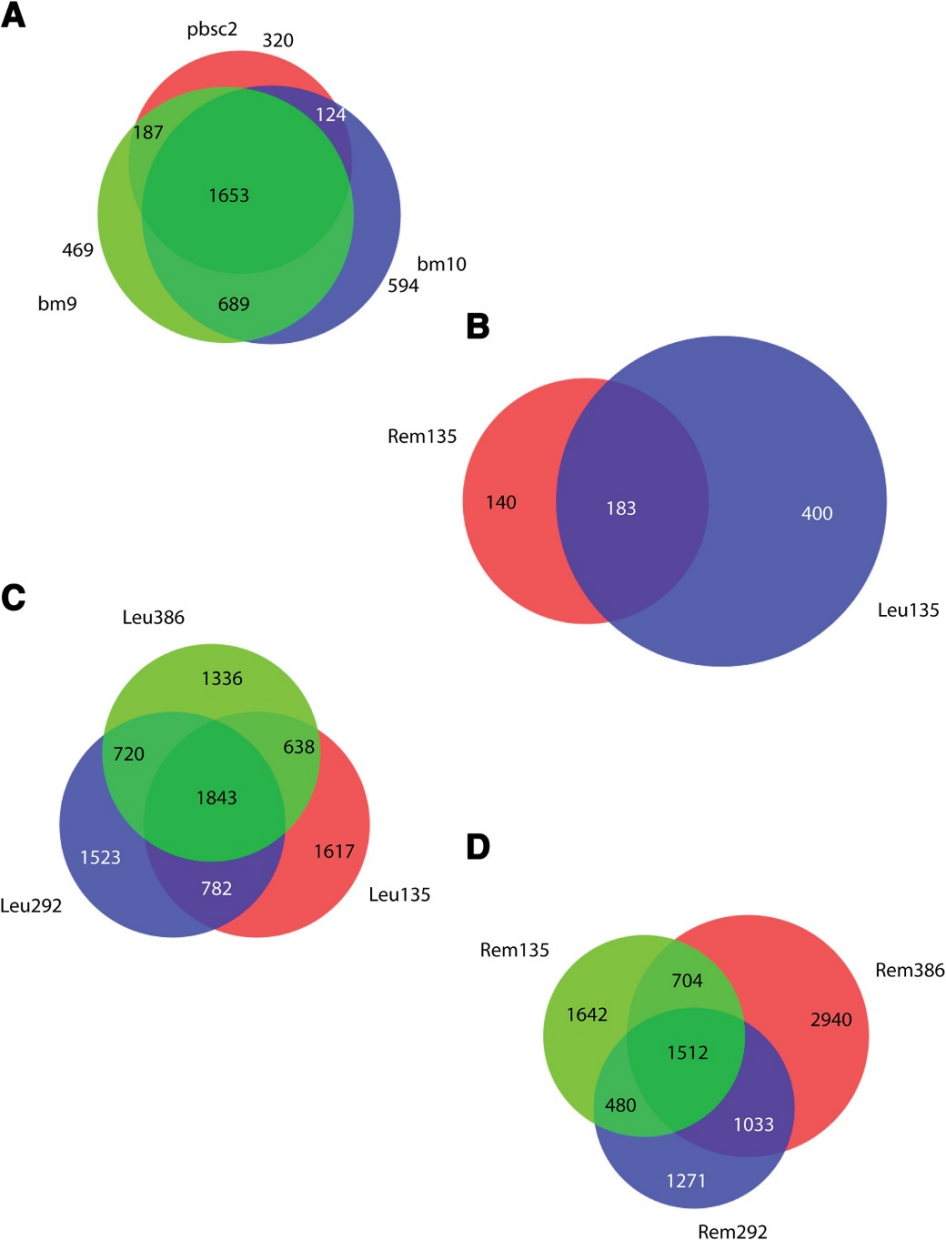
**Venn diagrams summarising peak region overlaps between samples analysed in this study.** Overlapping peak regions are shown after MACS peak analysis. **(A)** Peaks on chromosome 21 from three non-leukaemic individuals where CD19 cells were positively selected using FACS. A high degree of overlapping peaks were observed. **(B)** Peaks from matching leukaemic and remission samples from individual 135. Although there are some overlapping peaks (183), there are a substantial number of distinct peaks in each sample. **(C)** The extent of overlapping peaks between 3 leukaemic samples. **(D)** The extent of overlapping peaks between 3 remission samples.

We isolated four haematopoietic cell populations (CD34, CD19, CD33, CD45) at major stages of development corresponding to the arrested stages of development in paediatric leukaemia. This was achieved by positive selection using fluorescent-labelled antibodies and Fluorescent Activated Cell Sorting (FACS) from four individuals. This would enable us to track changes in DNA methylation between cell lineages and contrast them with leukaemic cells. After MACS enrichment peak analysis, a large proportion of peaks were common between the CD19 cells from three individuals, confirming the premise of tissue-specific DNA methylation profiles in haematopoietic cells (Figure 1A).

When comparing DNA methylation enrichment peaks between leukaemic and remission samples (tumour versus normal) from the same individual, distinct enrichment peaks are seen; these are likely to correlate to disease state (Figure 1B). The number of overlapping peaks between leukaemic and remission samples were fewer compared to the haematopoietic cell analyses (Figure 1C and 1D) and could be indicative of the difference in sample qualities.

For each of the samples analysed in this study, we have generated track hubs that can be uploaded and visualised on the UCSC Genome Browser. This permits the immediate visualisation of regions of differential DNA methylation with potential biological significance. Moreover, we have performed Infinium analysis on these samples, and visualisation using the Genome Browser permits direct comparison to other publicly available data such as The Cancer Genome Atlas (TCGA) [9] and TARGET (Therapeutically Applicable Research to Generate Effective Treatments) [10]. This also permits further analysis and comparison to publicly available data using the Galaxy [11,12] and Cistrome [13] web servers.

In summary, our data represent one of the first DNA methylation enrichment analyses using SOLiD™MBD-Seq on archival bone marrow smears from children diagnosed with ALL. Such specimens are readily available in most pathology laboratories across the world and are amenable to genomic-scale analysis, as we have demonstrated here. These data should prove valuable for other DNA methylation studies in childhood ALL in haematopoeitic cell development.

## Availability of supporting data

Supporting data is available from the GigaScience Database, GigaDB [14] and at NCBI under BioProject PRJNA272864.

## Data file details

- SRA Files included BioProject PRJNA272864
- MACS and HOMER output files of peaks and peak locations
- Track Hubs for UCSC Genome Browser

## Abbreviations

ALL: Acute lymphoblastic leukaemia
HOMER: Hypergeometric Optimization of Motif EnRichment
MACS: Model-based Analysis for ChIP-Seq
MBD: Methyl-binding domain

## References

[1] Serre D, Lee B, Ting A. MBD-isolated Genome Sequencing provides a high-throughput and comprehensive survey of DNA methylation in the human genome. Nucleic Acids Res. Jan 2010;38(2):391–9. doi: 10.1093/nar/gkp992. http://www.ncbi.nlm.nih.gov/pubmed/19906696. 10.1093/nar/gkp992 PMC2811030 19906696

[2] Voullaire L, Saffery R, Davies J, Earle E, Kalitsis P, Slater H (1999). Trisomy 20p resulting from inverted duplication and neocentromere formation. Am J Med Genet.

[3] Wong NC, Ashley D, Chatterton Z, Parkinson-Bates M, Ng H-K, Halemba MS (2012). A distinct DNA methylation signature defines pediatric pre-B cell acute lymphoblastic leukemia. Epigenetics : Official J DNA Methylation Soc..

[4] Zhang Y, Liu T, Meyer CA, Eeckhoute J, Johnson DS, Bernstein BE (2008). Model-based analysis of ChIP-Seq (MACS). Genome Biol.

[5] Heinz S, Benner C, Spann N, Bertolino E, Lin YC, Laslo P (2010). Simple Combinations of Lineage-Determining Transcription Factors Prime cis-Regulatory Elements Required for Macrophage and B Cell Identities. Molecular Cell.

[6] HOMER. Homer. http://homer.salk.edu/homer/chipseq/.

[7] Aplenc R, Orudjev E, Swoyer J, Manke B, Rebbeck T. Leukemia : Official J Leuk Soc Am Leuk Res Fund, UK. 2002; 16(9):1865–6. 10.1038/sj.leu.2402681 12200706

[8] Chatterton Z, Morenos L, Mechinaud F, Ashley DM, Craig JM, Sexton-Oates A (2014). Epigenetic deregulation in pediatric acute lymphoblastic leukemia.. Epigenetics : Official J DNA Methylation Soc..

[9] Weinstein JN, Collisson EA, Mills GB, Shaw KRM, Ozenberger BA, Ellrott K (2013). The Cancer Genome Atlas Pan-Cancer analysis project. Nat Genet.

[10] TARGET. Target.https://ocg.cancer.gov/programs/target.

[11] Giardine B, Riemer C, Hardison RC, Burhans R, Elnitski L, Shah P (2005). Galaxy: a platform for interactive large-scale genome analysis. Genome Res.

[12] Galaxy. Galaxy. https://usegalaxy.org/.

[13] Liu T, Ortiz JA, Taing L, Meyer CA, Lee B, Zhang Y (2011). Cistrome: an integrative platform for transcriptional regulation studies. Genome Biol.

[14] Wong NCL, Meredith GD, Marnellos G, Dudas M, Parkinson-Bates M, Halemba MS, et al. Supporting material for: Paediatric Leukaemia DNA methylation profiling using MBD enrichment and SOLiD Sequencing on archival bone marrow smears. GigaScience Database. http://dx.doi.org/10.5524/100099. 10.1186/s13742-015-0050-0 PMC4364676 25789165

